# Age‐associated microRNA expression in human peripheral blood is associated with all‐cause mortality and age‐related traits

**DOI:** 10.1111/acel.12687

**Published:** 2017-10-17

**Authors:** Tianxiao Huan, George Chen, Chunyu Liu, Anindya Bhattacharya, Jian Rong, Brian H. Chen, Sudha Seshadri, Kahraman Tanriverdi, Jane E. Freedman, Martin G. Larson, Joanne M. Murabito, Daniel Levy

**Affiliations:** ^1^ The Framingham Heart Study Framingham MA USA; ^2^ The Population Sciences Branch Division of Intramural Research National Heart, Lung and Blood Institute National Institutes of Health Bethesda MD USA; ^3^ Department of Computer Science and Engineering University of California San Diego CA USA; ^4^ Department of Biostatistics Boston University School of Public Health Boston MA USA; ^5^ Longitudinal Studies Section Translational Gerontology Branch Intramural Research Program National Institute on Aging National Institutes of Health Bethesda MD USA; ^6^ Department of Medicine Section of General Internal Medicine Boston University School of Medicine Boston MA USA; ^7^ Department of Medicine University of Massachusetts Medical School Worcester MA USA

**Keywords:** aging, cardiometabolic traits, methylation, microRNA, mortality, mRNA

## Abstract

Recent studies provide evidence of correlations of DNA methylation and expression of protein‐coding genes with human aging. The relations of microRNA expression with age and age‐related clinical outcomes have not been characterized thoroughly. We explored associations of age with whole‐blood microRNA expression in 5221 adults and identified 127 microRNAs that were differentially expressed by age at *P *<* *3.3 × 10^−4^ (Bonferroni‐corrected). Most microRNAs were underexpressed in older individuals. Integrative analysis of microRNA and mRNA expression revealed changes in age‐associated mRNA expression possibly driven by age‐associated microRNAs in pathways that involve RNA processing, translation, and immune function. We fitted a linear model to predict ‘microRNA age’ that incorporated expression levels of 80 microRNAs. MicroRNA age correlated modestly with predicted age from DNA methylation (*r *=* *0.3) and mRNA expression (*r *=* *0.2), suggesting that microRNA age may complement mRNA and epigenetic age prediction models. We used the difference between microRNA age and chronological age as a biomarker of accelerated aging (Δage) and found that Δage was associated with all‐cause mortality (hazards ratio 1.1 per year difference, *P *=* *4.2 × 10^−5^ adjusted for sex and chronological age). Additionally, Δage was associated with coronary heart disease, hypertension, blood pressure, and glucose levels. In conclusion, we constructed a microRNA age prediction model based on whole‐blood microRNA expression profiling. Age‐associated microRNAs and their targets have potential utility to detect accelerated aging and to predict risks for age‐related diseases.

## Introduction

Human aging is a complex process that has been linked to dysregulation of numerous cellular and molecular processes, including shortened telomere length (Harley *et al*., 1990), altered DNA damage response (Moskalev *et al*., 2013), loss of protein homeostasis (Tomaru *et al*., 2012), cellular senescence (Childs *et al*., 2015), and mitochondrial dysfunction (Green *et al*., 2011). Those cellular and molecular processes can lead to a variety of diseases including cancer, cardiovascular disease, and neurological disease, as well as an increased risk of mortality (Fontana *et al*., 2010; López‐Otín *et al*., 2013).

Recent studies have revealed that human aging can be characterized by changing patterns of DNA methylation (Hannum *et al*., 2013; Horvath, 2013) and expression of protein‐coding genes (Peters *et al*., 2015). A growing body of research suggests that aging is associated with changes in DNA methylation both genome‐wide and at specific C‐G dinucleotide (CpG) loci. Two recent studies developed age predictors based on the methylation state of CpGs in whole blood and other tissues (Hannum *et al*., 2013; Horvath, 2013). The resultant DNA methylation‐based predicted age (*i.e*., DNAm age) was associated with chronological age in several independent studies. The difference between DNAm age and chronological age (*i.e*., DNAm Δage) has been proposed as an index of accelerated aging and was reported to be associated with all‐cause mortality and several coronary heart disease risk factors (Marioni *et al*., 2015; Christiansen *et al*., 2016; Horvath *et al*., 2016).

At the mRNA level, a recent meta‐analysis of whole‐blood gene expression in ~15 000 individuals identified 1497 mRNAs that are differentially expressed in relation to age (Peters *et al*., 2015). An age predictor based on mRNA expression (*i.e*., mRNA age) highlighted genes involved in mitochondrial, metabolic, and immune function‐related pathways as key components of aging processes. The difference between mRNA age and chronological age (*i.e*., mRNA Δage) correlated with many metabolic risk factors including blood pressure, total cholesterol levels, fasting glucose, and body mass index (BMI) (Peters *et al*., 2015).

MicroRNAs (miRNAs) are a class of small noncoding RNAs that downregulate protein‐coding genes by either cleaving target mRNAs or suppressing translation of mRNAs into proteins (Lee & Ambros, 2001; Lee *et al*., 2004; Cordes & Srivastava, 2009). Research in a *Caenorhabditis elegans* model system revealed changes in miRNA expression in relation to lifespan and longevity (Boehm & Slack, 2005; Ibáñez‐Ventoso *et al*., 2006; Pincus *et al*., 2011). In humans, highly specific miRNA expression patterns are correlated with many age‐related diseases including cardiovascular disease (Small & Olson, 2011; Huan *et al*., 2015c) and cancer (Lu *et al*., 2005; Hayes *et al*., 2014). Recent studies have examined differentially expressed miRNAs in relation to age in whole blood (ElSharawy *et al*., 2012), peripheral blood mononuclear cells (PBMC) (Noren Hooten *et al*., 2010), and serum (Noren Hooten *et al*., 2013; Zhang *et al*., 2014). These studies, however, were based on small sample sizes, limiting the power to investigate age‐related changes in miRNA expression. We hypothesized, a priori, that it would be possible to create a miRNA signature of age that is predictive of chronological age and that age prediction based on miRNA expression is biologically meaningful and can be used as a biomarker of risk for age‐related outcomes including all‐cause mortality.

In a previous study, we measured miRNA expression in whole blood from more than 5000 Framingham Heart Study (FHS) participants. We investigated the heritability of miRNA expression and performed a genome‐wide association study (GWAS) of miRNA expression to identify miR‐eQTLs (Huan *et al*., 2015b). Our results showed that miRNAs are under strong genetic control. In the present study, we further investigated whole‐blood miRNA expression in relation to chronological age in FHS participants. Fig. S1 shows the overall study design. We identified 127 miRNAs that were differentially expressed in relation to chronological age, and performed internal validation by splitting the samples 1:1 into two independent sample sets. An integrative miRNA–mRNA coexpression analysis and miRNA target prediction revealed many age‐related pathways underlying age‐associated molecular changes. We also defined and evaluated an age predictor based on miRNA expression levels (*i.e*., miRNA age). Our results indicate that the difference between miRNA age and chronological age (*i.e*., miRNA Δage) is associated with multiple age‐related clinical traits including all‐cause mortality, coronary heart disease (CHD), hypertension, blood pressure, and glucose levels. In addition, we compared miRNA age with DNAm age and mRNA age in FHS participants.

## Results

### Study population

Table 1 shows the characteristics of the 5221 FHS participants in this study (2295 participants from the FHS offspring cohort and 2926 participants from the FHS third‐generation cohort). At the time of measured sample collection for miRNA isolation, the FHS Offspring cohort was on average 20 years older than the Third Generation cohort (mean age 66 vs. 46 years). As expected, given the age differences between cohorts, the Offspring cohort had a higher prevalence of cardiovascular disease risk factors. In addition, during 6 years of follow‐up, incident deaths occurred more commonly in the Offspring cohort (257 vs. 12).

**Table 1:** Framingham heart study offspring and third generation cohort study participant characteristics

Phenotypes/Covariates	Offspring cohort *N* = 2295	Third generation cohort *N* = 2926
Male (%)	44	46
Age (years), mean (SD)	66 (9)	46 (9)
Body mass index (kg m^−2^), mean (SD)	28.3 (5.4)	28.0 (5.9)
Systolic blood pressure (mm Hg), mean (SD)	129 (17)	116 (14)
Diastolic blood pressure (mm Hg), mean (SD)	73 (10)	74 (9)
Serum glucose (mg dL^−1^), mean (SD)	107 (23)	96 (18)
High‐density lipoprotein (mg dL^−1^), mean (SD)	59 (18)	60 (18)
Total cholesterol (mg dL^−1^), mean (SD)	186 (37)	187 (35)
Triglycerides (mg dL^−1^), mean (SD)	118 (70)	113 (79)
Current smokers, *n* (%)	147 (6)	341 (12)
Hypertension, *n* (%)	1449 (63)	719 (25)
Hypertension, no hypertensive treatment, *n* (%)	209 (9)	170 (6)
Prevalent diabetes mellitus, *n* (%)	317 (14)	156 (5)
Prevalent diabetes mellitus, no diabetes treatment, *n* (%)	108 (5)	56 (2)
Prevalent coronary heart disease, *n* (%)	219 (10)	31 (1)
Deaths, *n* (%)	257 (11)	12 (0)
Hypertensive treatment, *n* (%)	1196 (52)	544 (19)
Diabetes treatment, *n* (%)	209 (9)	103 (4)
Lipids treatment, *n* (%)	951 (41)	453 (15)

We split the 5221 FHS samples 1:1 by pedigrees into independent discovery and replication sets (see Methods). The mean age was 54.9 in the discovery set and 55.4 in the replication set (*P *=* *0.21). Other clinical factors did not differ between the discovery and replication sets.

### Identification of differentially expressed miRNAs in relation to chronological age

**Figure 1:**
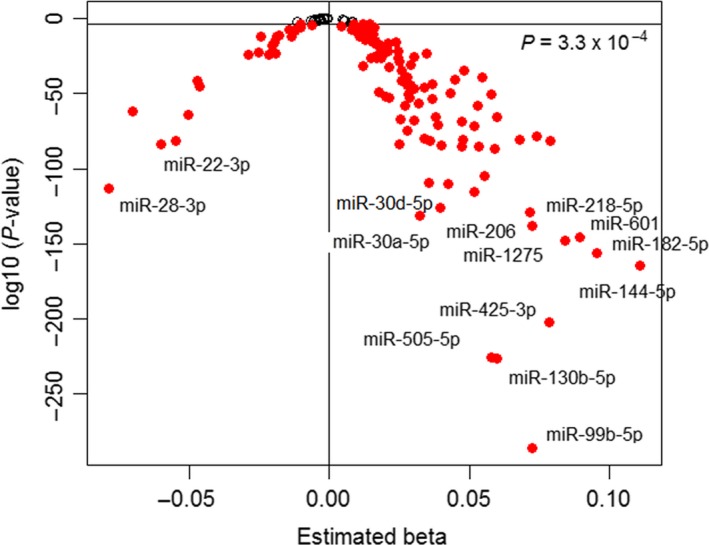
Volcano plot of differentially expressed miRNAs in relation to chronological age. Higher Ct values indicate lower miRNA expression levels. Therefore, positive beta values indicate negative associations between miRNA expression and age.

We identified 127 miRNAs that were differentially expressed in relation to chronological age at *P *<* *3.3 × 10^−4^ (Bonferroni‐corrected, 0.05/150). The incremental proportion of interindividual difference in age explained by the 127 differentially expressed miRNAs ranged from partial *r* ^*2*^ of 0.002–0.15. Table 2 provides results for the top 25 miRNAs, and Table S1 provides the full list. Of the 127 age‐associated miRNAs, 103 (81%) miRNAs were negatively correlated with age and 24 (19%) miRNAs were positively correlated (Fig. 1). Age‐related miRNA expression studies in whole blood (ElSharawy *et al*., 2012), PBMCs (Noren Hooten *et al*., 2010), and serum (Noren Hooten *et al*., 2013; Zhang *et al*., 2014) have similarly reported that the majority of age‐related miRNAs show decreased expression in older individuals.

**Table 2:** Top 25 differentially expressed miRNAs in relation to chronological age

miRNA	Estimated Beta^a^	SE	R‐squared	*P*‐Value
miR‐99b‐5p	0.07	0.002	0.15	1.16E‐286
miR‐130b‐5p	0.06	0.002	0.11	3.04E‐227
miR‐505‐5p	0.06	0.002	0.12	3.04E‐226
miR‐425‐3p	0.08	0.002	0.10	8.58E‐203
miR‐144‐5p	0.11	0.004	0.09	1.55E‐165
miR‐182‐5p	0.10	0.003	0.08	7.29E‐157
miR‐1275	0.08	0.003	0.08	5.40E‐149
miR‐601	0.09	0.003	0.08	4.48E‐146
miR‐206	0.07	0.003	0.08	4.48E‐139
miR‐30a‐5p	0.03	0.001	0.08	9.07E‐132
miR‐218‐5p	0.07	0.003	0.07	4.94E‐130
miR‐30d‐5p	0.04	0.002	0.07	9.10E‐127
miR‐502‐3p	0.05	0.002	0.06	5.28E‐116
miR‐28‐3p	−0.08	0.003	0.06	7.96E‐114
miR‐197‐3p	0.04	0.002	0.06	3.69E‐111
miR‐320b	0.04	0.002	0.07	4.77E‐110
miR‐576‐3p	0.06	0.002	0.06	2.58E‐105
miR‐181a‐5p	0.06	0.003	0.05	2.61E‐87
miR‐18a‐5p	0.05	0.003	0.05	2.93E‐86
miR‐223‐5p	0.05	0.002	0.05	6.86E‐86
miR‐339‐5p	0.04	0.002	0.05	1.65E‐85
miR‐24‐3p	0.03	0.001	0.05	2.08E‐84
miR‐22‐3p	−0.06	0.003	0.05	2.14E‐84
miR‐345‐5p	0.04	0.002	0.05	1.66E‐82
miR‐302c‐3p	0.08	0.004	0.05	2.83E‐82

a Higher Ct values indicate lower miRNA expression levels. Therefore, positive beta values indicate negative associations between miRNA expression and age.

The age associations of miRNAs in the discovery and replication sets were highly consistent with Pearson's correlations (*r*) of beta values (effect size) of > 0.99 and of log10‐transformed *P* values of 0.97 (Fig. S2). Ninety‐one percentage of differentially expressed miRNAs in relation to age in the discovery set (at *P *<* *3.3 × 10^−4^) replicated in the replication set (at *P *<* *3.3 × 10^−4^). The validation results suggest that age‐associated miRNA expression signatures are robust and highly replicable.

In conducting a sensitivity analysis, we identified differentially expressed miRNAs in relation to age separately in the FHS Offspring cohort and FHS Third Generation cohort (TablesS2and S3). At *P *<* *3.3 × 10^−4^, there were 32 age‐associated miRNAs identified in the FHS Offspring cohort and 57 in the larger FHS Third Generation cohort. A total of 23 miRNAs overlapped between cohorts. The Pearson correlation of beta values for all 150 measured miRNAs between cohorts was 0.67 (Fig. S3). These results suggest that age‐associated miRNA expression differs slightly across age groups.

### miRNA age prediction

We used elastic net regression (Friedman *et al*., 2010) to select miRNAs for building an age prediction model using 10‐fold cross‐validation. To ensure unbiased validation, the age prediction model was trained only in the discovery sample set. Table S4> reports the results of the prediction model that included 80 miRNAs. Seventy‐two of the 80 miRNAs in the prediction model showed differential expression in relation to age at *P *<* *3.3 × 10^−4^.

**Figure 2:**
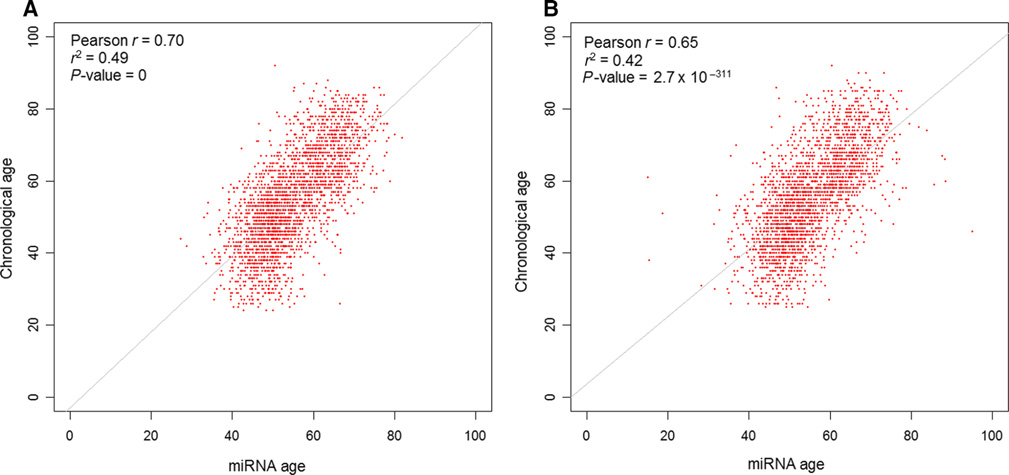
miRNA age vs. chronological age. (A) In the discovery set; (B) in the replication set.

The correlation between miRNA predicted age (miRNA age) and chronological age was significant in the discovery (Pearson's correlation *r *=* *0.70; *P < *1 × 10^−320^) and replication sets (*r *=* *0.65; *P = *2.7 × 10^−311^) (Fig. 2). We hypothesized that the difference between miRNA age and chronological age (miRNA Δage) is an index of ‘biological’ aging, with a positive Δage indicating accelerated aging, and a negative Δage reflecting slower aging in comparison with chronological age. miRNA Δage was not associated with miRNA age (Fig. S4).

In conducting a sensitivity analysis to account for cohort effects, we used the same method to train a miRNA age prediction model in the FHS Offspring and Third Generation cohorts separately (Fig. S5). When the two cohorts were used as separate discovery‐replication sets, the correlation between miRNA age and chronological age was stronger in the Offspring cohort (Pearson's correlation *r *=* *0.50 and *P *=* *1.2 × 10^−73^) than in FHS Third Generation cohort (Pearson's correlation *r *=* *0.36 and *P *=* *2.7 × 10^−45^). The different correlation between miRNA age and chronological age in cohort‐specific analysis could be explained by differences in mean age, age range, and unmeasured technical factors.

### Heritability of miRNA Δage

The estimate of heritability of miRNA Δage ($h_{\mathrm{miR}-\Delta \mathrm{age}}^{2}$) was 0.38. To determine whether any specific genetic variants correlate with miRNA Δage, we performed a genome‐wide association study of miRNA Δage using all available FHS samples (see Fig. S6 for the Manhattan plot). There were no SNPs associated with miRNA Δage at *P *<* *5 × 10^−8^.

We previously reported that miRNA expression levels are highly heritable, and identified 5269 miRNA expression‐associated SNPs for 76 miRNAs within +/‐ 1 MB of the associated SNP (*i.e*., *cis‐*miR‐eSNPs). We further explored whether miRNA Δage is linked to *cis‐*miR‐eSNPs. No *cis‐*miR‐eSNPs were associated with miRNA Δage at *P *<* *1 × 10^−5^.

### Influence of blood cell types on miRNA age

We compared the differentially expressed miRNAs in relation to age both with and without adjustment of blood cell types. As shown in Fig. S7, the Pearson correlation coefficients of beta values with vs. without adjustment for blood cell types were > 0.99, and for log10‐transformed P values, it was > 0.99. In addition, 122 of the 127 age‐associated miRNAs (96%) remained significant after adjusting for blood cell types, indicating that adjusting for blood cell type proportions had little effect on our results.

In conducting a sensitivity analysis adjusting for white blood cell counts, the miRNA age predictor exhibited a weaker but still high correlation with chronological age (*r *=* *0.61) in the replication set. Cell type effects explained a proportion of age variability, which may have resulted in the weaker correlation observed between miRNA age and chronological age after adjusting for cell types.

### miRNA Δage is predictive of all‐cause mortality and correlated with many metabolic traits

**Figure 3:**
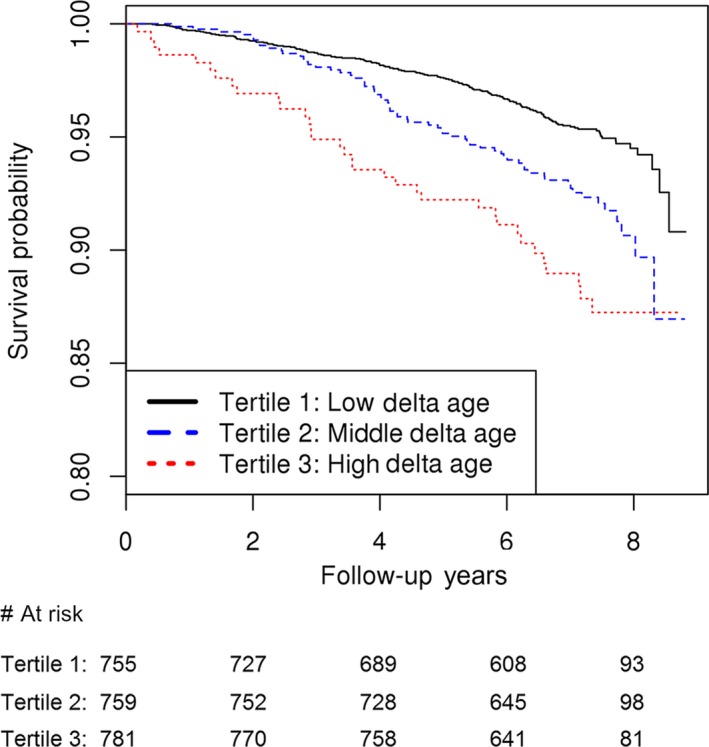
Survival probability by tertiles of miRNA Δage.

The association of miRNA Δage with all‐cause mortality was tested in FHS Offspring participants (there were too few deaths in the Third Generation cohort for meaningful analysis). We found that miRNA Δage was associated with mortality with a hazards ratio (HR) of 1.10 (95% CI 1.05–1.14; *P *=* *4.2 × 10^−5^) per year of miRNA Δage after adjustment for chronological age and sex. Kaplan–Meier survival curves for miRNA Δage tertiles are presented in Fig. 3. The plot illustrates higher mortality rates for those with higher Δage. Sensitivity analyses were performed to control for additional potential confounders (cigarette smoking, HDL cholesterol, total cholesterol, triglycerides, systolic and diastolic blood pressure, fasting blood glucose, BMI, lipid treatment, diabetes treatment, hypertension treatment, prevalent cancer, prevalent CHD, and prevalent diabetes). The fully adjusted HR was 1.15 (95% CI 1.08–1.22; *P *=* *2.5 × 10^−5^).

The association of miRNA Δage with prevalent CHD was tested in all available individuals (a total of 250 prevalent CHD cases). The associations of miRNA Δage with prevalent diabetes and hypertension were tested in individuals who were not receiving medications to treat diabetes and hypertension (including 164 diabetes cases and 379 hypertension cases), respectively. We found miRNA Δage to be positively associated with CHD (*P *=* *3.8 × 10^−17^) and hypertension (*P *=* *0.002), after adjustment for chronological age, sex, and BMI (Table 3).

**Table 3:** Associations of miRNA Δage with prevalent CHD, diabetes, hypertension, and cardiometabolic traits

Trait/Disease	Chronological age				miRNA Δage			
	Estimated Beta	SE	T‐Value	*P*‐Value	Estimated Beta	SE	T‐Value	*P*‐Value^a^
CHD	15.28	0.84	18.20	8.72E‐72	5.42	0.64	8.45	**3.76E‐17**
Hypertension	9.45	0.64	14.84	2.76E‐48	1.62	0.53	3.05	**2.34E‐03**
Diabetes	8.42	1.05	8.02	1.28E‐15	1.01	0.77	1.30	1.94E‐01
Total cholesterol	0.82	0.05	15.84	3.81E‐54	−0.05	0.07	−0.82	4.14E‐01
High‐density lipoprotein	0.07	0.02	2.73	6.34E‐03	0.06	0.03	2.00	4.61E‐02
Triglycerides	0.26	0.10	2.51	1.21E‐02	−0.18	0.13	−1.36	1.73E‐01
Systolic blood pressure	0.55	0.02	26.27	5.09E‐136	0.10	0.03	3.78	**1.58E‐04**
Diastolic blood pressure	0.08	0.01	6.00	2.24E‐09	0.06	0.02	3.65	**2.67E‐04**
Glucose	0.31	0.02	15.81	5.78E‐54	0.07	0.02	2.86	**4.30E‐03**
Body mass index	0.004	0.01	0.52	6.01E‐01	−0.002	0.01	−0.24	8.11E‐01

a The *P*‐value threshold for significance for miRNA Δage vs. traits analyses (*P *<* *0.005) was determined by the Bonferroni method (0.05/10). Significant *P*‐values are shown in boldface.

We also tested the associations of miRNA Δage with multiple cardiometabolic traits using all available FHS participants who were not receiving medications to treat hypertension, dyslipidemia, or diabetes (*N* = 2993). These analyses revealed miRNA Δage to be positively associated with systolic blood pressure, diastolic blood pressure, and fasting glucose levels at *P *<* *0.005 (Bonferroni‐corrected, 0.05/10 tests) after adjusting for age, sex, and BMI (Table 3).

### miRNA–mRNA coexpression, miRNA targets, and pathway analyses

To better understand how miRNAs might contribute to aging processes, we further tested whether expression levels of age‐associated mRNA transcripts are mirrored by differential expression of age‐associated miRNAs. For this purpose, we analyzed miRNA–mRNA coexpression in the same set of FHS individuals (*n* = 5012) in whom miRNA and mRNA expression data were both available. For the 127 age‐associated miRNAs, the coexpression analysis identified 4682 mRNAs that were highly coexpressed with the 123 age‐related miRNAs at FDR < 0.05, including 24 310 miRNA–mRNA coexpression pairs. Of the 4682 mRNAs that were coexpressed with age‐associated miRNAs, 551 mRNAs were previously reported to be age‐associated (Peters *et al*., 2015); the total number of reported age‐associated mRNAs in that study was 1497. Comparison of the two ratios (4682/17 318 measured mRNAs and 551/1497) yielded *P *<* *1 × 10^−16^ (by the hypergeometric test), suggesting that age‐associated differences in miRNA expression are indicative of age‐associated changes in their coexpressed mRNAs. Gene ontology enrichment analysis (Table S5) showed that the coexpressed mRNAs are enriched for translation (*P *=* *3.3 × 10^−7^), immune response (*P *=* *9.4 × 10^−7^), and RNA processing (*P *=* *1.7 × 10^−5^).

Among the miRNA–mRNA coexpression pairs, TargetScan v7.0 (Lewis *et al*., 2005; Agarwal *et al*., 2015) predicted 3552 of the 4682 mRNAs to be potential corresponding targets for the coexpressed age‐associated miRNAs, including 406 of the 551 age‐associated mRNAs (~74%). Details of miRNAs and their coexpressed/predicted target mRNAs involved in each GO category are provided in Table S6.

### Comparing miRNA age with mRNA age and DNAm age

**Figure 4:**
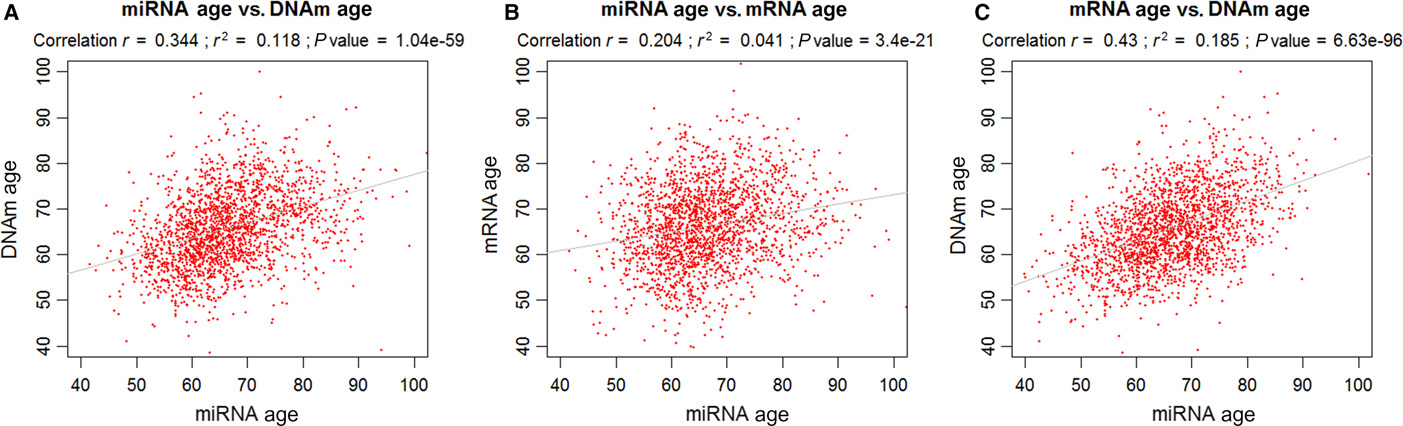
Comparison of miRNA Δage, mRNA age, and DNAm age. (A) miRNA age vs. DNAm age; (B) miRNA age vs. mRNA age; (C) mRNA age vs. DNAm age.

In our previous studies, we used mRNA expression and DNA methylation data to predict age (*i.e*., mRNA age and DNAm age) in FHS Offspring participants (Marioni *et al*., 2015; Peters *et al*., 2015). In order to compare miRNA age with mRNA age and DNAm age, we used miRNA age calculated only in the FHS Offspring cohort. The Pearson correlation (*r)* of chronological age with miRNA age in the FHS Offspring cohort was 0.50 (*P = *1.2 × 10^−73^). The *r* values of chronological age with mRNA age and DNAm age were 0.56 and 0.73, respectively. As shown in Fig. 4, miRNA age was positively correlated with mRNA age and DNAm age, with *r* values of 0.20 and 0.34, respectively. DNAm age and mRNA age were also positively correlated (*r *=* *0.43).

When mRNA age and DNAm age were both regressed on chronological age, the *r* ^*2*^ was 0.57. Regressing miRNA age, mRNA age, and DNAm age on chronological age yielded an *r* ^*2*^ of 0.63. Using data from individuals whose miRNA and mRNA data were both available (*N* = 2784), we further tested the associations of miRNA Δage with cardiometabolic traits adjusting for mRNA Δage, as shown in Table S7. The results did not change appreciably when adding mRNA Δage in the model. The miRNA Δage remained associated with CHD, hypertension, BP, and glucose. We did not include DNAm Δage in the model, because DNA methylation data were available in a smaller number of eligible individuals.

## Discussion

We systematically assessed age‐associated differences in whole‐blood miRNA expression levels and developed a miRNA age predictor. We calculated the difference between miRNA predicted age and chronological age to generate Δage, which we put forth as an indicator of an individual's rate of biological aging. We found Δage to be associated with risk for all‐cause mortality and with prevalent CHD, hypertension, and glucose levels.

In comparison with previous age‐related miRNA expression studies (Noren Hooten *et al*., 2010, 2013; ElSharawy *et al*., 2012; Zhang *et al*., 2014), our study included > 25 times as many participants (*N* = 5221 vs *N* < 200) and a wide age range (24–92 years). As such, our study was well powered to investigate differential levels of miRNA expression in relation to age. Among the 150 whole‐blood miRNAs available for analysis in this study, we identified 127 miRNAs that were differentially expressed in relation to age at *P *<* *0.05/150. The differential expression patterns of miRNAs were highly consistent between separate discovery and replication sample sets. The effect sizes (*r* ^*2*^) of individual differential miRNA expression with age were small, ranging from 0.05–0.15 (Table 2), limiting the use of single miRNAs as clinical biomarkers of age. The small effect sizes required a large sample size to identify a‐multiple miRNA age‐related biomarker.

By comparing the FHS Offspring and Third Generation cohorts, as shown in Fig. S3, the estimated beta values for 150 measured miRNAs in relation with age are consistent between cohorts. All 23 significant age‐associated miRNAs that overlapped between the two cohorts showed a concordant direction of effects. However, there were more than 50 miRNAs that were significant in the overall sample set, but not in either cohort. Because the cohort‐specific analysis reduced the available sample size by about half, the differences by cohort may have arisen from reduced power. Another possible reason for differences by cohort is that the correlation of miRNA expression with age may differ in the younger and older cohorts, which differed in age by an average of 20 years (Noren Hooten *et al*. 2013). However, we cannot exclude undetected technical factors contributing to differences between the cohorts.

Many of our identified miRNAs were previously shown to be differentially expressed in relation to age in whole blood (ElSharawy *et al*., 2012), PBMCs (Noren Hooten *et al*., 2010), and serum (Noren Hooten *et al*., 2013; Zhang *et al*., 2014; Smith‐Vikos *et al*., 2016). For example, miR‐130b‐5p (the second most significant miRNA in our study) was previously reported to be negatively associated with age in serum (Zhang *et al*., 2014). In addition, miR‐340‐3p was reported to be significantly downregulated in long‐lived individuals vs. short‐lived individuals (Smith‐Vikos *et al*., 2016). In mouse studies, miR‐130b‐5p was shown to regulate cholesterol and triglyceride homeostasis (Wagschal *et al*., 2015). One of our top 25 miRNAs, miR‐24, was reported to be negatively correlated with age in whole blood (ElSharawy *et al*., 2012) and PBMCs (Noren Hooten *et al*., 2010). One study found that increased expression of miR‐24 in T‐cell lines downregulated the expression of histone H2A family member X, which plays a key role in DNA damage response (Brunner *et al*., 2012). We also found that most of the age‐associated miRNAs are downregulated in older individuals, consistent with previous findings.

miRNAs play a pivotal role in post‐transcriptional regulation of protein‐coding genes. Therefore, we hypothesized that age‐associated mRNAs might be regulated by age‐associated miRNAs. Our miRNA–mRNA coexpression and target prediction analysis revealed that the coexpressed/targeted mRNAs for the identified age‐associated miRNAs were enriched for previously reported age‐associated mRNAs (enrichment *P *<* *1.0 × 10^−16^) (Peters *et al*., 2015). This result suggests that age‐associated changes in miRNA expression levels may alter the expression of their targeted protein‐coding genes, which play vital roles in aging processes. GO analysis results were consistent with effects on known aging mechanisms including regulation of transcription, translation, and immune response.

The regulation of transcription and translation of protein‐coding genes is essential to aging processes. In *C. elegans* studies, reducing the levels of key proteins involved in translation, such as ribosomal proteins, ribosomal‐protein S6 kinase, and translation initiation factors, increased the lifespan of *C. elegans* (Hansen *et al*., 2007; Pan *et al*., 2007). Ribosomal‐protein S6 kinase also regulates lifespan in mammalian models (Selman *et al*., 2009). miRNAs in *Drosophila* have been shown to block the eIF4F translation initiation complex assembly, thereby inhibiting overall translation of protein‐coding genes (Fukaya *et al*., 2014). Similarly, a recent study reported that miR‐139 represses the translation initiation factor EIF4G2 and thereby reduces overall protein synthesis (Emmrich *et al*., 2016). Our results found that many miRNAs (*e.g.,* miR‐139 and miR‐140) were coexpressed with and targeted many ribosomal genes (*e.g., RPL11* and *RPL30*) as well as translation initiation and elongation genes (*e.g., EEF2* and *EIF4B*) (Table S6). Our results call for further functional studies to explore the specific mechanisms by which age‐associated miRNAs exert their effects on aging and age‐related diseases through regulation of key transcription and translation genes.

Many miRNAs have been found to be involved in immune pathways. For example, previous studies showed that miR‐181a regulates local immune balance (Liu *et al*., 2012) and T‐cell sensitivity (Li *et al*., 2007), although its mechanistic action remains unclear. We found that miR‐181a‐3p was coexpressed with and predicted to target three immune response genes, namely *CXCL16*,* RAB27A,* and *SPON2*. *RAB27A* is also involved in the T‐cell activation pathway. Other identified miRNAs such as miR‐193b‐3p target immune function‐related genes, including *CTSW*,* ETS1*,* FAIM3*,* ICOS*,* TGFBR3*,* DPP4*, and *KIF13B*. Similarly, miR‐31‐5p targets *CD27*,* FASLG*,* INPP5D*,* TCF7*,* TGFBR3*, and *DPP4* and may merit further investigation.

Heritability analysis revealed that miRNA Δage is a heritable trait (*h* ^*2*^=0.38). We did not, however, find genome‐wide significant associations with miRNA Δage, likely due to an insufficient sample size for GWAS of this trait.

We also showed that individuals with a predicted miRNA age greater than their chronological age (*i.e.,* higher miRNA Δage) exhibit higher blood pressure and glucose levels, a higher prevalence of CHD and hypertension, as well as increased risk of death from all causes. In comparison, DNAm Δage was previously shown to be associated with all‐cause mortality, but not with prevalent CHD or hypertension (Marioni *et al*., 2015; Horvath *et al*., 2016). mRNA Δage was reported to be associated with blood pressure, glucose, and HDL and total cholesterol, but not with all‐cause mortality (Peters *et al*., 2015). In comparing miRNA age with DNAm age and mRNA age in FHS Offspring participants, miRNA age showed modest correlations with DNAm age (*r *=* *0.3) and mRNA age (*r *=* *0.2), suggesting that miRNA age may complement mRNA and epigenetic age prediction models and can capture unique aspects of the molecular mechanisms of aging and age‐related diseases.

One limitation of this study is that we assessed miRNA expression in whole blood, which consists of multiple cell types and plasma. To address this, we tested whether the proportions of individual blood cell types influenced the association of age with miRNA expression levels. In comparing results with and without cell type adjustment, we observed only minimal changes due to cell type composition. In our previous miRNA expression QTL study, we also did not find substantial cell type effects on the correlation between genetic variants and miRNA expression. Another limitation is that we were unable to perform external replication of our results. To our knowledge, no other study has published extensive analyses of miRNA expression in relation to age in a large sample size with a wide age range. In addition, by splitting our sample set into independent discovery and replications sets, we demonstrated a high degree of replicability of our age‐related miRNAs.

## Experimental procedures

### Study population

The FHS Offspring cohort was initially recruited in 1971 and included 5124 offspring (and their spouses) from the FHS Original cohort. The FHS Third Generation cohort was recruited from 2002 to 2005 and included 4095 adult children of the Offspring cohort participants (Feinleib *et al*., 1975; Splansky *et al*., 2007). The samples used for miRNA analysis in the present study (*N* = 5221) included 2295 participants from the FHS Offspring cohort who attended the eighth examination cycle (Exam 8, 2005–2008) and 2926 participants from the FHS Third Generation cohort who attended the second examination cycle (Exam 2, 2008–2011). Because the samples from the Offspring and Third Generation cohorts share familial relatedness, we merged the samples from the two cohorts together. Next, the entire FHS sample set was split 1:1 by pedigrees into separate discovery and replication sets. To ensure that the samples in the discovery set did not share relatedness with samples in the replication set, a given pedigree was assigned either to the discovery or to the replication set, but not in both.

Gene (mRNA) expression data were obtained from 2446 FHS Offspring (Exam 8) and 3180 Third Generation (Exam 2) participants (Huan *et al*., 2015a). DNA methylation data were obtained from 2377 FHS Offspring cohort participants (Exam 8). miRNA and mRNA expression data were available for 5012 participants, and there were 2079 participants with miRNA, mRNA, and DNA methylation data. All participants provided written consent for genetic research.

### miRNA expression profiling and normalization

Peripheral whole‐blood samples were collected in PAXgene tubes (Asuragen, Inc., Austin, TX, USA). Purified RNA was extracted using the PAXgene Blood RNA System Kit (Qiagen, Venlo, Netherlands). The WT‐Ovation Pico RNA Amplification System (NuGEN, San Carlos, CA, USA) was used to amplify 50 ng RNA samples according to the manufacturer's recommended protocols. RNA quality was measured using an Agilent 2100 Bioanalyzer and RNA concentration was quantified using a NanoDrop ND‐1000 spectrophotometer. miRNA profiling was carried out at the high‐throughput Gene Expression and Biomarker Core Laboratory at the University of Massachusetts Medical School using TaqMan chemistry‐based miRNA assays by using Dynamic Arrays on BioMark System (Fluidigm, South San Francisco, CA, USA).

The initial miRNA list encompassed all TaqMan miRNA assays available at the start of the study, including 754 miRNAs that were profiled in ~600 FHS individuals. 346 miRNAs expressed in > 20% samples were further profiled in 2445 FHS Offspring and 3245 Third Generation cohort participants. Quantification of miRNA expression was based on cycle threshold (Ct), where lower Ct values signify higher miRNA expression levels. miRNAs with Ct values ≥ 27 indicated that they were not expressed in the sample. Outlier miRNAs with Ct values ≥ 5 standard deviations from the mean Ct value were categorized as missing. We excluded miRNAs expressed in < 5000 samples and samples with > 10% of miRNAs having missing values from analysis. A total of 150 miRNAs and 5221 samples remained for analysis, and 1.2% of remaining missing values were replaced with Ct = 27.

As previously described, raw miRNA Ct values were adjusted for four technical variables: isolation batch (50 batches), RNA concentration, RNA quality (defined as RNA integrity number [RIN]), and RNA 260/280 ratio (ratio of absorbance at 260 and 280 nm; measured using a spectrophotometer). This normalization model explained 20% to 60% of variability of raw miRNA measurements for 80% of miRNAs.

### mRNA expression profiling

Messenger RNA (mRNA) expression profiling of whole blood‐derived RNA was performed using the Affymetrix Human Exon 1.0 ST GeneChip platform including 17 318 transcripts. Robust multichip average (RMA) methods were used to normalize mRNA expression values (log‐2‐transformed expression intensity) with quality control measures as previously described (Joehanes *et al*., 2013).

### DNA methylation

Buffy coat preparations were obtained from peripheral whole‐blood samples. Genomic DNA was extracted from buffy coat using the Gentra Puregene DNA extraction kit (Qiagen, Venlo, Netherlands) and bisulfite‐converted using EZ DNA Methylation kit (Zymo Research, Irvine, CA, USA). Samples underwent whole‐genome amplification, fragmentation, array hybridization, and single base pair extension. DNA methylation quantification was conducted in two laboratory batches at the Johns Hopkins Center for Inherited Disease Research (lab batch #1, *N* = 576) and University of Minnesota Biomedical Genomics Center (lab batch #2, *N* = 2270).

Methylation beta values were generated using the DASEN methodology implemented in the wateRmelon package in R version 3.0, which includes background adjustment and quantile normalization. Sample exclusion criteria included poor SNP matching of control positions, missing rate > 1%, poor single nucleotide polymorphism (SNP) matching to the 65 SNP control probe locations, outliers from multidimensional scaling (MDS), and sex mismatch. Probes were excluded if missing rate > 20%, previously identified to map to multiple locations, or having an underlying SNP (minor allele frequency > 5% in European ancestry (EUR) 1000 Genomes Project data) at the CpG site or within 10 bp of the single base extension. A total of 2377 samples and 443 252 CpG probes remained for analysis.

### Imputing cell counts

The cell count proportions of whole blood were measured in 2138 Third Generation FHS participants (Exam 2), but not for all samples used in this study. Cell counts were imputed using a partial least‐squares regression method (Abdi, 2010) applied to mRNA expression. The estimated cell count proportion values imputed were generally consistent with the measured values in the 2138 samples with cross‐validated estimates of prediction accuracy *r* ^2* *^> 0.8 for white blood cell, red blood cell, platelet, lymphocyte percent, monocyte percent, and eosinophil percent, and *r* ^*2*^ = 0.25 for basophil percent. miRNA expression analysis accounting for cell counts effects was performed in the 5012 samples.

### Identifying differentially expressed miRNAs in relation to age

A linear mixed‐effects model was used to model miRNA expression Ct values (150 miRNAs in total) as the dependent variable and chronological age as an explanatory variable, adjusting for sex, technical variables (batch, RNA concentration, RNA quality score, and 260/280 ratio), and family structure. In a sensitivity analysis, additional adjustments were made for imputed cell counts. The statistical analysis was implemented in the *lmekin()* R function (http://cran.r-project.org/web/packages/kinship/) (Almasy & Blangero, 1998). Correcting for 150 tests (the number of miRNAs), Bonferroni‐corrected *P *<* *3.3 × 10^−4^ was used as the significant threshold.

### miRNA expression age prediction

The standardized residuals of miRNA expression Ct values (150 miRNAs) were obtained by adjusting raw Ct values for three technical covariates (RNA concentration, RNA quality score, and 260/280 ratio) and sex. Because miRNA expression measurements for the FHS Offspring and Third Generation cohorts were performed in independent batches, we did not adjust Ct values for batch effect. In a sensitivity analysis, additional adjustments were made for imputed cell counts.

We used an elastic net regression model (implemented in the R package *glmnet* function (Friedman *et al*., 2010)) to regress chronological age on miRNAs. Elastic net regression is a combination of traditional Lasso and ridge regression methods, emphasizing model sparsity while appropriately balancing the contributions of coexpressed miRNAs. To ensure an unbiased validation, the prediction model was trained in the discovery set. Optimal regularization parameters were estimated via 10‐fold cross‐validation. The alpha parameter of *glmnet* was set to 0.5, and the lambda value from the best prediction model was set to 0.2. *glmnet* automatically selected miRNAs for building an age predictor and reported effect size for each miRNA. miRNA predicted age (*i.e*., miRNA age) was calculated in the replication set using the predictor trained in the discovery set. miRNA Δage was defined as miRNA age minus chronological age.

### Estimation of the additive heritability of miRNA Δage

The narrow‐sense heritability estimate of miRNA Δage (denoted as $h_{\mathrm{miR}-\Delta \mathrm{age}}^{2}$ was the proportion of the additive polygenic genetic variance of the total phenotypic variance of miRNA Δage: $h_{\mathrm{miR}-\Delta \mathrm{age}}^{2}=\sigma_{A}^{2}/\sigma_{\mathrm{miR}-\Delta \mathrm{age}}^{2}$, where $\sigma_{A}^{2}$ denotes the additive polygenic genetic variance and $\sigma_{\mathrm{miR}-\Delta \mathrm{age}}^{2}$ denotes the total phenotypic variance of a gene expression trait. The $h_{\mathrm{miR}-\Delta \mathrm{age}}^{2}$ estimate was obtained using variance‐component methodology implemented in the *lmekin()* function of Kinship Package in R (Almasy & Blangero, 1998). Heritability estimation for miRNA Δage was performed using all FHS samples.

### All‐cause mortality ascertainment

Outcome analyses included all deaths that occurred prior to January 1, 2014 (about 6 years of follow‐up). Survival status was ascertained using multiple strategies, including routine contact with participants for health history updates, surveillance at the local hospital, review of obituaries in the local newspaper, and queries to the National Death Index. We requested death certificates, hospital and nursing home records prior to death, and autopsy reports. When cause of death was undeterminable, the next of kin were interviewed. The date and cause of death were reviewed by an endpoint panel of three investigators.

Associations between miRNA Δage and mortality were tested using Cox proportional hazards regression models utilizing the *coxph()* function in the ‘survival’ R library (https://stat.ethz.ch/R-manual/R-devel/library/survival/html/coxph.html), adjusting for age at sample collection and sex. Potential cofounders that were included in the fully adjusted model included systolic blood pressure, diastolic blood pressure, BMI, HDL cholesterol, total cholesterol, triglycerides, fasting blood glucose, smoking status, lipid treatment, diabetes treatment, hypertension treatment, prevalent cardiovascular disease, prevalent cancer, and prevalent diabetes. Hazard ratios for miRNA Δage were expressed as annual risk of death over 6 years of follow‐up. Survival curves were drawn by tertiles of miRNA Δage.

### Association analysis of miRNA Δage with prevalent CHD, prevalent diabetes, prevalent hypertension, and cardiometabolic traits

To avoid the cofounding effects of drug treatment, all association tests of miRNA Δage and cardiometabolic traits were performed in individuals who were not receiving antihypertensive, lipids, or diabetes treatment (*n* = 2993). Linear mixed‐effects models were used to test associations between miRNA Δage (dependent variable) and cardiometabolic traits (independent variable), including systolic blood pressure, diastolic blood pressure, total cholesterol, HDL cholesterol, triglycerides, fasting blood glucose, and BMI using the *lmekin()* R function. All association tests were adjusted for chronological age, sex, and familial relatedness. To account for the effects of obesity on cardiovascular disease and other traits, we additionally adjusted for BMI (except in analyses of BMI).

Linear mixed‐effects models (R package *lmekin()* function) were used to test associations between miRNA Δage (dependent variable) and prevalent CHD, diabetes, and hypertension (independent variable, coding ‘1’ for cases and ‘0’ for controls), adjusting for age, sex, BMI, and familial relatedness. Association analysis of miRNA Δage with CHD was performed in all available samples (*n* = 5221, including 250 prevalent CHD cases). Diabetes and hypertension analyses were performed in individuals who were not receiving antidiabetic treatment (*n* = 4909, including 164 type‐II diabetes cases) and antihypertensive treatment (*n* = 3481, including 379 hypertension cases), respectively.

### miRNA–mRNA coexpression analysis

The coexpression analysis was performed on FHS participants in whom miRNA and mRNA data were both available. Linear mixed‐effects models (R package *lmekin()* function) were used to conduct pairwise coexpression analyses for all profiled mRNAs (*N* = 17 318) and 150 miRNAs, with fixed effects including age, sex, technical covariates, imputed cell types, surrogate variables (SV), and a random effect to account for family structure. As described above, the mRNA expression was quantified by log‐2‐transformed expression intensities. For miRNA expression, higher Ct values reflect lower miRNA expression levels. Adjustment was made for technical covariates (11 for mRNA expression and four for miRNA expression). Surrogate variables (SVs) were computed from the mRNA expression data using the R package ‘SVA’ (Leek & Storey, 2007), and 51 SVs associated with at least one miRNA at Bonferroni‐corrected *P *<* *3.3 × 10^−4^ (0.05/150) were included in the statistical model. The Benjamini–Hochberg method was used to compute false discovery rate (FDR). Significant miRNA–mRNA coexpression pairs were selected using FDR < 0.05.

### Predicting miRNA targets

For the coexpressed miRNA–mRNA pairs, we used TargetScan v7.0 (Lewis *et al*., 2005; Agarwal *et al*., 2015) to predict whether the mRNAs were the corresponding targets for the miRNAs. TargetScan predicts mRNA targets of miRNAs by searching for the presence of 8‐mer, 7‐mer, and 6‐mer sites that match the seed region of each miRNA. The sequences from 3′UTR, 5′UTR, and coding regions of each mRNA were downloaded from the University of California Santa Cruz (UCSC) Table Browser (https://genome.ucsc.edu/). The miRNA seed regions were downloaded from miRbase v21 (http://www.mirbase.org/).

### Genome‐wide association study of miRNA Δage

DNA was isolated from buffy coat or from immortalized lymphoblast cell lines in FHS participants. Genotyping was conducted with the Affymetrix 500K mapping array and the Affymetrix 50K gene‐focused MIP array, using previously described quality control procedures (Levy *et al*., 2009). Genotypes were imputed to the 1000 Genomes Project panel of approximately 36.3 million variants using MACH (Li *et al*., 2010). We filtered out SNPs with MAF < 0.05 and imputation quality ratio < 0.3 (the imputation quality ratio is denoted by the ratio of the variances of the observed and the estimated allele counts). About 9 million variants remained after the filter. The association of miRNA Δage with each SNP was tested by a linear mixed‐effects model that was implemented in the *lmekin()* R function. miRNA Δage was used as dependent variable, and each SNP as an explanatory variable adjusting for chronological age and sex.

### Gene ontology and pathway enrichment analysis

Coexpressed or predicted targeted mRNAs for age‐associated miRNAs were combined as gene sets and classified using Gene Ontology (GO) databases to identify potentially relevant biological processes. Fisher's exact test was used to calculate enrichment *P* values. DAVID Bioinformatics resources 6.7 (https://david.ncifcrf.gov/) (Huang *et al*., 2009), an online tool for GO analysis, was used for this analysis. The significant threshold was set at FDR < 0.05.

### Data access

The microRNA expression, mRNA expression, and DNA methylation data used in this article are available online in dbGaP (http://www.ncbi.nlm.nih.gov/gap; accession number phs000007).

## Authors’ contributions

D.L. and J.M.M. designed, directed, and supervised the project. T. H., G.C., and D.L. drafted the manuscript. M.G.L. directed and supervised the statistical analyses. T.H., C.L., J.R., and A.B. conducted the analyses. J.E.F. and K.T. conducted the miRNA expression experiments. All authors participated in revising and editing the manuscripts. All authors have read and approved the final version of the manuscript.

## Funding

The Framingham Heart Study is funded by National Institutes of Health contract N01‐HC‐25195 and HHSN268201500001I. The laboratory work for this investigation was funded by The Division of Intramural Research, National Heart, Lung, and Blood Institute, National Institutes of Health. The analytical component of this project was funded by the Division of Intramural Research, National Heart, Lung, and Blood Institute, and The Center for Information Technology, National Institutes of Health, Bethesda, MD and R56AG029451 (M.G.L.). The views expressed in this manuscript are those of the authors and do not necessarily represent the views of the National Heart, Lung, and Blood Institute; the National Institutes of Health; or the U.S. Department of Health and Human Services.

## Conflict of interests

The authors declare that they have no conflict of interests.

## Supporting information


**Fig. S1** Analysis flowchart.
**Fig. S2** Effect size and *P* values of differentially expressed miRNAs in relation to chronological age in the discovery and replication sets.
**Fig. S3** Effect size and *P* values of differentially expressed miRNAs in relation to chronological age in the FHS Offspring and Third Generation sets.
**Fig. S4** miRNA Δage vs. miRNA age.
**Fig. S5** miRNA age vs. chronological age.
**Fig. S6** Manhattan plot of genome‐wide associations with miRNA Δage
**Fig. S7** Comparison of effect size and *P* value of differentially expressed miRNAs in relation to chronological age before and after cell type adjustment.

**Table S1** Differentially expressed miRNAs in relation to chronological age at Bonferroni.
**Table S2** Differentially expressed miRNAs in relation to chronological age in the FH.
**Table S3** Differentially expressed miRNAs in relation to chronological age in the FHS Th.
**Table S4** miRNA age prediction formula.
**Table S5** Gene ontology results.
**Table S6** Coexpressed miRNAs and mRNAs in each GO category.
**Table S7** Associations of miRNA Δage with prevalent CHD, diabetes, hypertension, and cardiometabolic traits, adjusting for mRNA Δage
