# Supplementary material for: Age‐associated microRNA expression in human peripheral blood is associated with all‐cause mortality and age‐related traits
Source: Aging Cell. 2017 Oct 17;17(1):e12687. doi: 10.1111/acel.12687 (PMC5770777; doi:10.1111/acel.12687)
Supplement: Supplementary file 1 — Fig. S1 Analysis flowchart. Fig. S2 Effect size and P values of differentially expressed miRNAs in relation to chronological age in the discovery and replication sets. Fig. S3 Effect size and P values of differentially expressed miRNAs in relation to chronological age in the FHS Offspring and Third Generation sets. Fig. S4 miRNA Δage vs. miRNA age. Fig. S5 miRNA age vs. chronological age. Fig. S6 Manhattan plot of genome‐wide associations with miRNA Δage Fig. S7 Comparison of effect size and P value of differentially expressed miRNAs in relation to chronological age before and after cell type adjustment. [file ACEL-17-na-s001.docx]

**Supplementary Figures**

**Supplementary Figure 1: Analysis flowchart.**

**Supplementary Figure 2: Effect size and P values of differentially expressed miRNAs in relation to chronological age in the discovery and replication sets.** A) Comparison of effect size; B) Comparison of P values.

**A B**

**
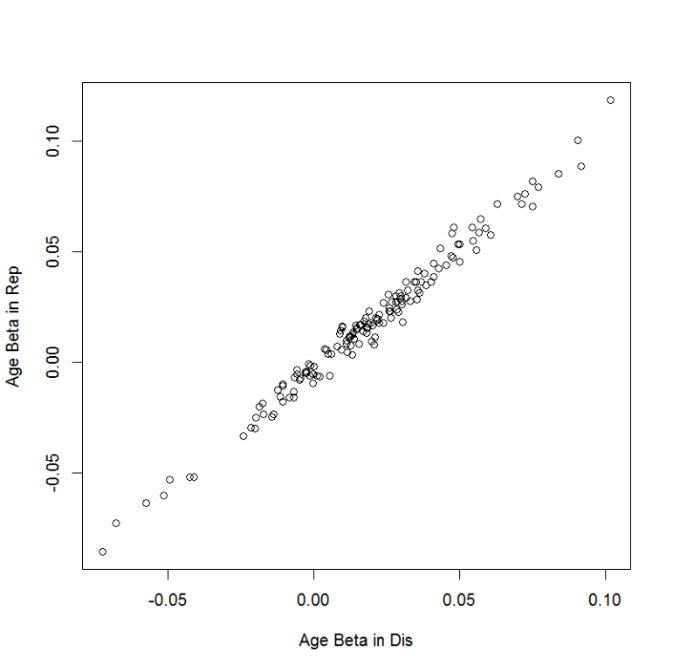

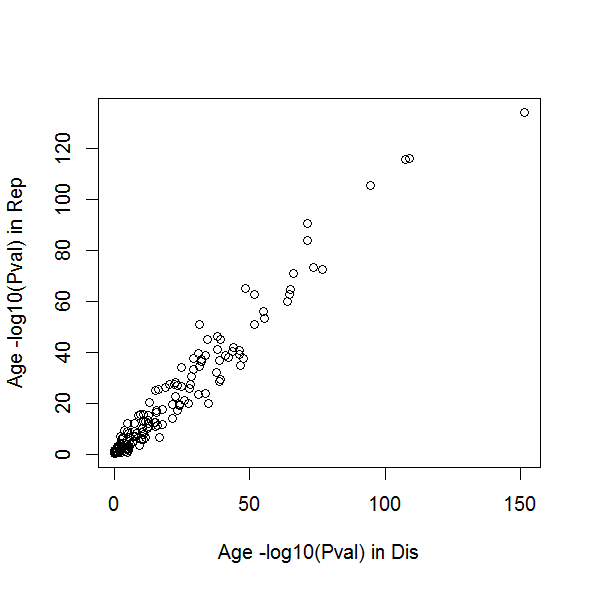
**

**Supplementary Figure 3: Effect size and P values of differentially expressed miRNAs in relation to chronological age in the FHS Offspring and Third Generation sets.** A) Comparison of effect size; B) Comparison of P values.

**A B**

**
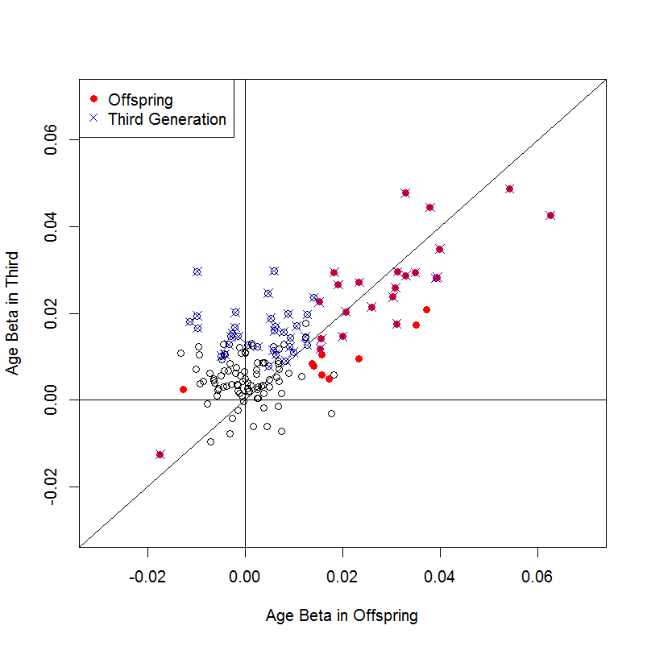

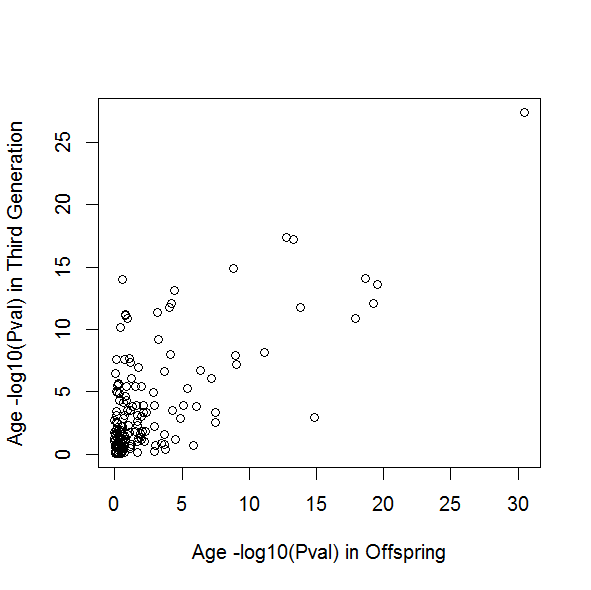
**

**Supplementary Figure 4: miRNA Δage versus miRNA age.** In the replication set, the Pearson correlation of miRNA Δage and miRNA age is 0.06.

**
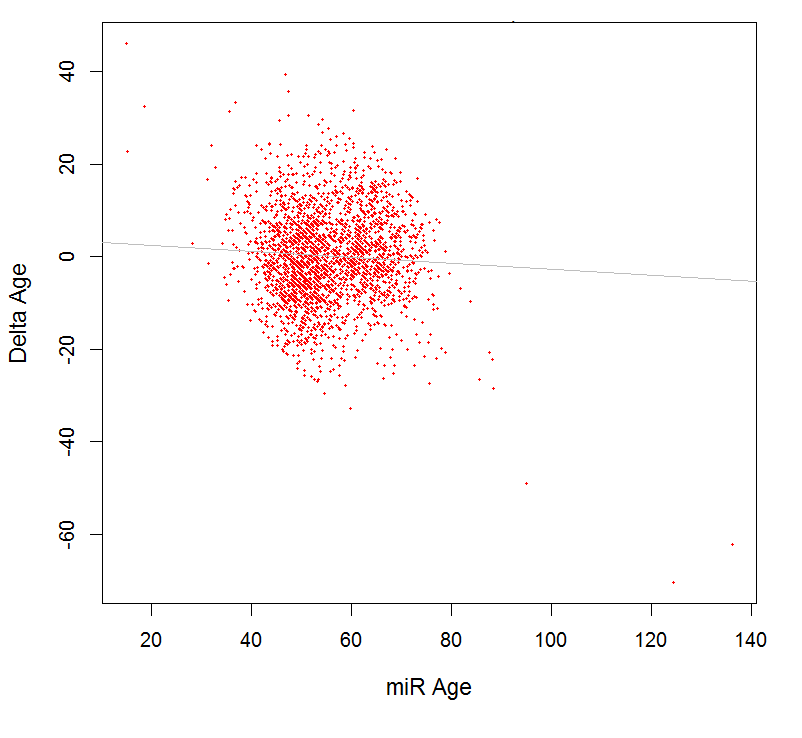
**

**Supplementary Figure 5: miRNA age versus chronological age.** A) In the FHS Offspring cohort; B) In the FHS Third Generation cohort.

**A B**

**
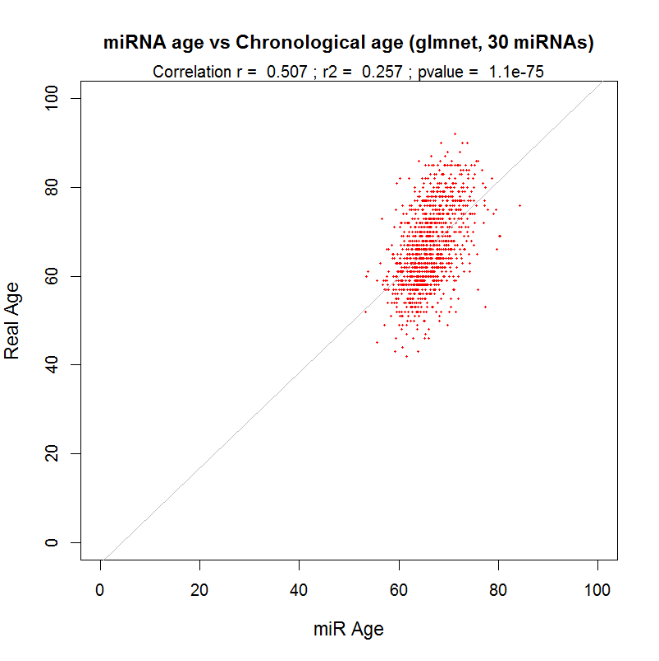

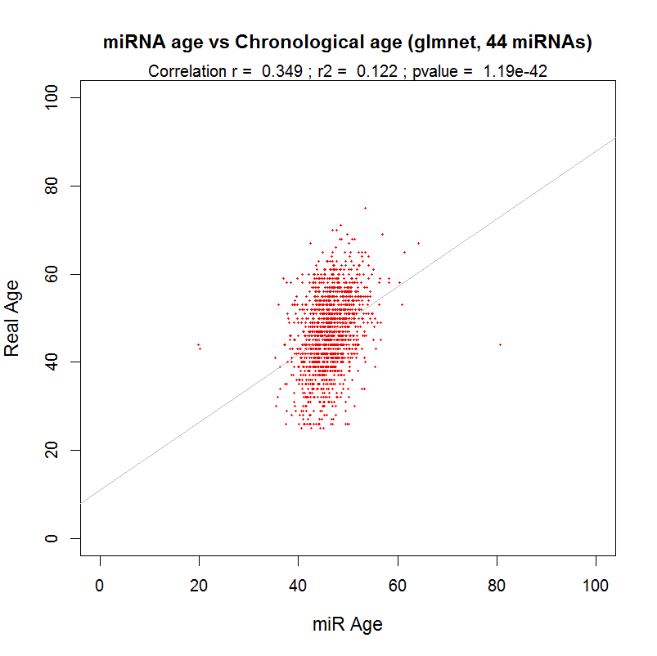
**

**Supplementary Figure 6: Manhattan plot of genome-wide associations with miRNA Δage**

**
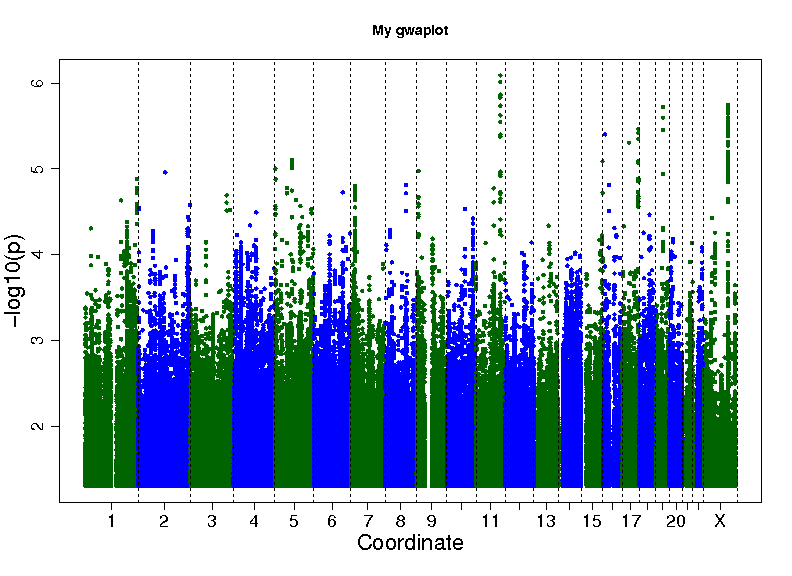
**

**Supplementary Figure 7: Comparison of effect size and P value of differentially expressed miRNAs in relation to chronological age before and after cell type adjustment.** A) Comparison of effect size; B) Comparison of P values.

**A B**

**
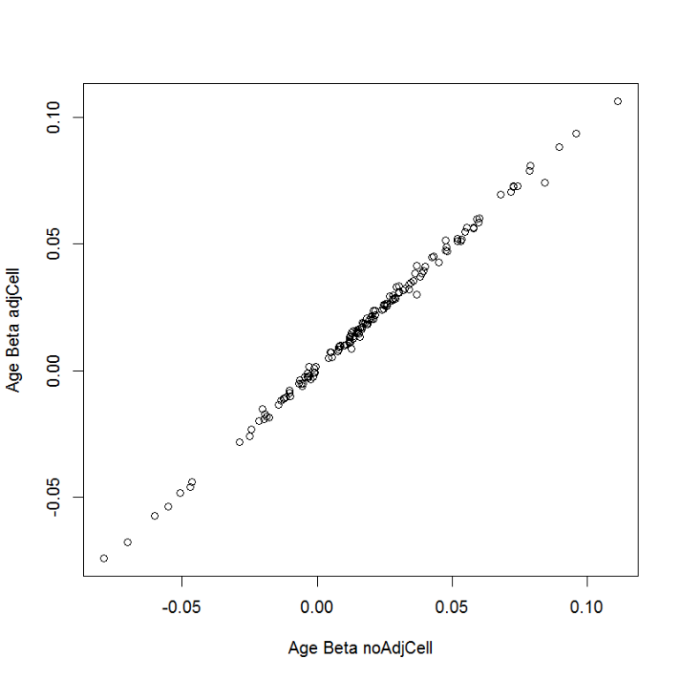

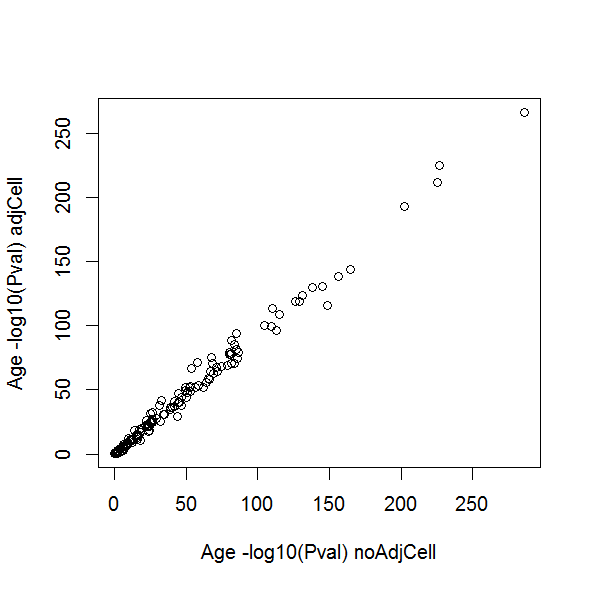
**
